# Role of stem/progenitor cells in reparative disorders

**DOI:** 10.1186/1755-1536-5-20

**Published:** 2012-12-27

**Authors:** Thavaneetharajah Pretheeban, Dario R Lemos, Benjamin Paylor, Regan-Heng Zhang, Fabio M Rossi

**Affiliations:** 1 The Biomedical Research Centre, The University of British Columbia, Vancouver, BC, V6T 1Z3, Canada

**Keywords:** Fibrosis, Fatty degeneration, Heterotopic ossification, Tissue specific stem cells, Mesenchymal stromal cells, TGFβ, BMP, Wnt

## Abstract

Adult stem cells are activated to proliferate and differentiate during normal tissue homeostasis as well as in disease states and injury. This activation is a vital component in the restoration of function to damaged tissue via either complete or partial regeneration. When regeneration does not fully occur, reparative processes involving an overproduction of stromal components ensure the continuity of tissue at the expense of its normal structure and function, resulting in a “reparative disorder”. Adult stem cells from multiple organs have been identified as being involved in this process and their role in tissue repair is being investigated. Evidence for the participation of mesenchymal stromal cells (MSCs) in the tissue repair process across multiple tissues is overwhelming and their role in reparative disorders is clearly demonstrated, as is the involvement of a number of specific signaling pathways. Transforming growth factor beta, bone morphogenic protein and Wnt pathways interact to form a complex signaling network that is critical in regulating the fate choices of both stromal and tissue-specific resident stem cells (TSCs), determining whether functional regeneration or the formation of scar tissue follows an injury. A growing understanding of both TSCs, MSCs and the complex cascade of signals regulating both cell populations have, therefore, emerged as potential therapeutic targets to treat reparative disorders. This review focuses on recent advances on the role of these cells in skeletal muscle, heart and lung tissues.

## Review

### Introduction

Tissue repair post-injury or during disease culminates in either complete restoration of tissue integrity, defined here as regeneration, or in a process that leads to the generation of stromal structures that replace functional tissue. These structures, while vital in ensuring tissue continuity, do not support, and in some instances even interfere with, tissue or organ function. The establishment of these stromal scars is referred herein as “repair”, and conditions in which they become predominant are called “reparative disorders”. This term encompasses diseases or symptoms exhibited during the repair process of damaged tissues that have been described in the literature since the early nineteenth century, including adipocyte accumulation (fatty degeneration), ectopic bone formation and fibrous tissue deposition [1-3]. In the context of mammalian biology, tissue regeneration is an essential process for restoring structure and function of traumatized organs. Regeneration of tissues is typically accompanied by acute or chronic inflammation caused by the disease or trauma, and involves the coordinated interaction among multiple cell types, including tissue specific stem/ progenitor cells (TSCs), mesenchymal stromal cells (MSCs) and immune cells. Many of the same cell types involved in regeneration also contribute to repair, suggesting that aberrant environmental cues and alterations of the signaling networks between these cells are central to the establishment of reparative disorders [4].

Several sources have been proposed for the progenitors involved in reparative disorders, including local sources, such as the damaged tissues themselves, and systemic sources, such as the bone marrow [5]. Locally, both tissue specific stem cells and ubiquitous mesenchymal and endothelial progenitors have been implicated in the development of the cellular effectors of repair: fibrotic matrix producing cells, adipocytes and osteocytes. Systemically, a role has been proposed for bone marrow derived cells reaching the repairing tissues through the bloodstream. Here, we review the evidence concerning these different types of stem cells and, in particular, the role of TSCs and MSCs in reparative disorders. We also provide an overview of the signaling pathways mediating their interactions.

The three most commonly occurring outcomes of reparative disorders are fibrosis, fatty degeneration and heterotopic ossification. Fibrosis is a defining characteristic in most reparative disorders and can take place in nearly every tissue. In fibrosis, damaged structures are gradually replaced by collagen-rich connective tissue resulting in anatomical anomalies as well as reduced functional capacities. The poorly defined fibroblast or myofibroblast, the effector cell components of connective tissue, is thought to be responsible for producing excess collagen and other extracellular matrix (ECM) proteins [6]. These same processes, however, also take place during normal regeneration, and are likely to be critical for its success. Another type of common reparative disorder is the accumulation of fat in damaged tissues leading to “fatty degeneration” and loss of function [4-6]. Usually, fat is found in newly formed adipocytes infiltrating the tissue, most often associated with concurrent fibrotic matrix deposition, and in these cases it is clearly associated with injuries and defective repair processes [7-9]. Finally, heterotopic ossification, also known as ectopic bone formation, is another frequent reparative complication that takes place in the context of excessive trauma, surgery, wounds and burns. While non-hereditary and hereditary extra-skeletal bone formation is discussed in detail elsewhere [10,11], here we will focus only on the source of osteogenic cells in aberrant repair processes.

### Tissue resident stromal cells

In the adult organism, fibroblasts, adipocytes and osteocytes are thought to be generated from the same multipotent mesenchymal progenitors. These progenitors, officially termed mesenchymal stromal cells (MSCs) in a positional paper from the International Society for Cellular Therapeutics [12] continue to be referred to as mesenchymal stem cells [13], despite the lack of clear experimental evidence supporting their ability to self-renew and satisfy the most basic definition of a stem cell [14]. The accumulation of these mature cell types in tissues that have failed to properly regenerate suggests that alterations in the function of MSCs may represent a common thread underlying reparative disorders [15]. While a minimal set of markers defining an MSC has been agreed upon [12], expression of these markers is clearly heterogeneous both *in vivo* and *in vitro* and does not currently allow their prospective purification. In addition, while MSCs retain a similar developmental potential in most tissues, the expression of specific markers may vary depending on their specific anatomical location, a reality that has hindered the proper characterization of stromal progenitors. To date, the most reliable characteristic of stromal progenitors is the ability to produce fibroblastic colonies and, under the appropriate culture conditions, to differentiate in adipocytes, chondrocytes and osteogenic cells. The generation of additional cell types, such as endothelium and skeletal muscle, has been reported, but no consensus exists on whether such expanded developmental potential is actually observed in physiological conditions. Indeed, in the absence of specific markers to identify them *in situ*, elucidating the role of MSCs in the maintenance of differentiated tissues, such as bone and fat depots *in vivo*, has been difficult and their importance is as yet unclear. Another role for this cell type that has emerged over the years has spurred their therapeutic exploitation in *ex vivo* delivery approaches, and lies in their ability to provide trophic support to multiple cell types following tissue damage.

**Figure 1:**
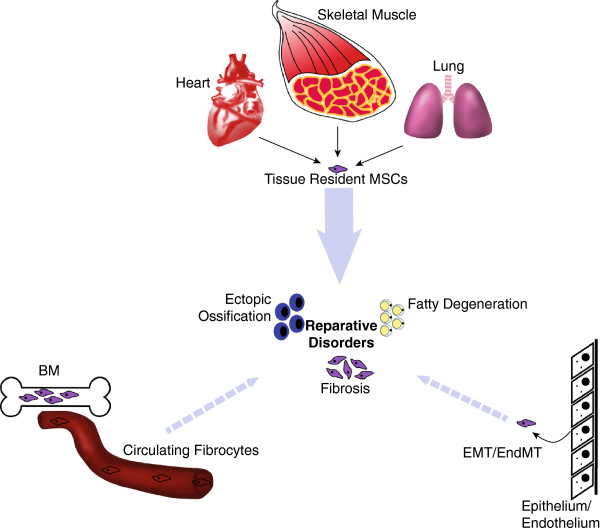
**Potential sources of MSCs in tissue repair.** During injury or disease tissue-resident MSCs (mesenchymal stromal/stem cells) can expand and provide trophic support for regeneration and/or differentiate to produce fibrosis, fatty degeneration or ectopic ossification or a combination of these. In addition, contribution to the fibrogenic cell pool by circulating “fibrocytes” originated from BM (bone marrow) and either EMT/EndMT (epithelial-mesenchymal transition/endothelial-mesenchymal transition) are proposed even though their existence, as well as the impact of their contribution to the deposition of fibrotic matrix, is controversial.

Almost all postnatal organs and tissues contain MSCs [16], and the list of resident stromal cells involved in tissue homeostasis and repair now includes multiple cell types, such as pericytes in multiple tissues [17,18], fibro-adipogenic progenitors (FAPs) in muscle and adipose tissues [19,20], adipose precursor cells in skin [21] and myo-fibroblasts in the liver, kidneys and lungs [22]. It is unclear whether these are truly distinct cell types, or if they, rather, represent a diffused stromal progenitor system comprised of cells that display different propensity to spontaneously differentiate along specific lineages, but possess a common underlying developmental potential that can be revealed by exposure to the appropriate stimuli. In addition to these ubiquitous progenitors, tissue fibroblasts have also been proposed to arise from circulating bone marrow cells or, in specific organs, from epithelial-mesenchymal transition (EMT) (see reviews [23,24]); the controversial evidence supporting these claims will be discussed below. The multiple proposed sources of fibrogenic cells in adult life are schematically depicted in Figure 1.

Despite the uncertainties and controversies stemming from their incomplete characterization, recent literature clearly supports a role for MSCs not only in immunomodulation, trophic support, angiogenesis and other processes associated with successful tissue regeneration, but also in reparative disorders, such as fibrosis and fatty degeneration [25-27]. Here we discuss recent advances on the role of MSCs in skeletal and cardiac muscle as well as lung reparative disorders.

### The role of tissue-resident MSCs and TSCs in reparative disorders

#### Skeletal muscle – an ideal regenerative/degenerative model system

**Figure 2:**
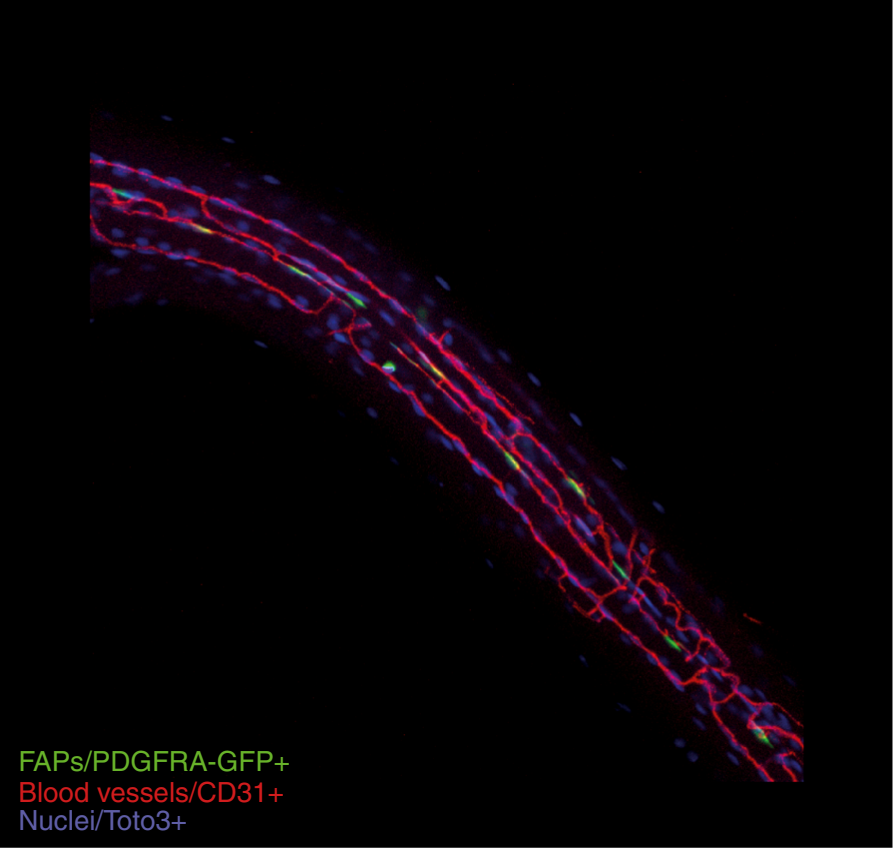
**Fibro/adipogenic progenitors (FAPs) in skeletal muscle.** Confocal image of a cluster of muscle fibers harvested from non-damaged muscle showing the relationship between mesenchymal progenitors expressing nuclear GFP under the control of the PDGFRα locus and fiber-associated blood vessels positive for CD31 (red). Nuclei are stained blue.

Skeletal muscle, like many other organs, contains stromal cells that are active following injury in both healthy animals and disease models. In addition, stromal cells are believed to play an essential role in muscle development [28]. These stromal cells are found in the muscle interstitium as well as associated with blood vessels (Figure 2). While often found in a perivascular position, they have been reported not to express typical pericytic markers, such as NG2 [29,30]. In mice, these cells are capable of spontaneously differentiating along the fibrogenic and adipogenic lineages *in vitro*, and have, therefore, been provisionally called fibro-adipogenic progenitors (FAPs) [19]. FAPs can be isolated as CD45^-^/CD31^-^/α7 /CD34^+^/Sca-1^+^/PDGFRα^+^ cells. Cells expressing fibroblast markers (ER-TR7/FSP1/αSMA) or adipogenic markers, such as perilipin, arise from individual multipotent progenitors contained in this population. We and others [19,31] have further demonstrated that the fate of these progenitors is heavily dependent on the environment within which they reside. This local microenvironment dictates whether these cells provide trophic support to satellite cells, the endogenous myogenic stem cells, to yield complete regeneration of injured muscle or whether they generate the components of the fibro-fatty tissue infiltrates often found in degenerating muscle tissue. A role for these cells in the efficient regeneration of muscle is also supported by depletion experiments relaying on the expression of CRE recombinase under the control of the transcription factor Tcf4 [28]. This approach led to the depletion of only about 40% of the cells, and highly efficient deletion of stromal progenitors has yet to be achieved in any organ. More recently, also in support of a paracrine effect of MSCs, Lavasani *et al.*[32] reported that muscle derived stem/progenitor cells (MDSPCs), essentially stromal cells isolated from young mice, were able to improve degenerative changes in aged mice and observed a correlation between MDSPC abundance and better muscle fiber maturation post injury.

Skeletal muscle resident FAPs are quiescent in healthy tissue, but quickly respond to damage by entering proliferation and expanding to infiltrate the extracellular space between myofibers, where they presumably carry out their trophic support function. Following this period of expansion, and at a time in which myogenic progenitors are differentiating to regenerate myofibers, the excess FAPs generated during the expansion phase quickly disappear and the cells return to quiescence. This, however, is not the case in degenerative disease or during aging. In situations where regeneration fails, FAPs persist and generate fibrous/fatty tissue that, while maintaining structural integrity, hinders function and subsequent regeneration [33,34]. It is important to note that FAPs have been reported to be the only source of collagen producing cells in regenerating skeletal muscle [35], clearly pointing to these cells as the main origin of fibrosis. While strong support exists for both the trophic role of FAPs and their role on tissue degeneration, the signals regulating their growth, survival and differentiation are still unknown. As these signals represent promising therapeutic targets for the treatment of acute and chronic injuries, they are the objects of intense investigation [36].

#### Role of skeletal muscle derived MSCs in heterotopic ossification

Heterotopic ossification is a common finding following severe or repeated soft tissue injuries, usually associated with fibrotic or fibro-fatty degeneration. The involvement of MSC-like progenitors in this process is supported by observations in war-traumatized patients, whose muscle are found to contain cells capable of producing fibroblastic colonies and to give rise to osteoblasts, adipocytes and/or chondrocytes [37-39]. Presumably in these instances, while a subset of local mesenchymal progenitors proliferate and differentiate into fibroblasts producing fibrotic matrix, some adopt a different lineage commitment and become osteoprogenitors. They, in turn, differentiate into osteoblasts, eventually resulting in ectopic bone formation [40]. Indeed, while FAPs were originally described as bipotent progenitors due to their spontaneous differentiation along the fibrogenic and adipogenic lineages, recent evidence strongly supports their role in ectopic bone formation. Following this, early reports suggested that these cells could generate alkaline phosphatase positive cells [31]. More recently, [41] reported that CD31^–^/CD45^–^/PDGFRa^+^/Sca-1^+^/Tie2^+^ progenitors from skeletal muscle could generate cells expressing the osteoprogenitor marker osterix when exposed to bone morphogenic protein 2 (BMP2) *in vitro* or in a transplantation setting. In addition, lineage-tracing experiments showed that these cells were the main source of ectopic cartilage and bone when BMP2 was delivered to skeletal muscle *in vivo*[41]. In these experiments, not all osteogenic cells expressed the Tie2-CRE activated lineage-tracing marker. However, it is unclear whether this reflects inefficient CRE mediated recombination or the participation of Tie2 negative cells in this process. Supporting our previous results, without addition of BMP2, these cells failed to adopt the fate of cartilage or bone, demonstrating that environmental cues are dictating the destiny of these progenitors and, thereby, the outcome of wound healing.

#### Role of tissue-specific stem cells in skeletal muscle degeneration

Although the role of MSCs in the development of reparative disorders in skeletal muscle has been clearly demonstrated, there is also evidence implicating a role of TSCs in modulating aberrant repair processes in this organ. The importance of the principal stem cell in adult myogenesis, the satellite cell, in regeneration is well established, but the relationship between these stem cells and tissue degeneration is much more complex and not well understood. Satellite cells reside between the myofibers and basement membrane of the muscle bundle [42] and unlike tissues that experience constant wear and tear, these TSCs are normally quiescent/stable, and are not activated until prompted by injury. Quiescent satellite cells are identified by their expression of Pax7, a paired homeobox transcription factor partly responsible for survival and specification of the myogenic cell lineage [43]. Although an excellent marker of all satellite cells in the wild-type adult, Pax7 is only required during the neonatal stage for satellite cell maintenance, proliferation and differentiation [44,45]. Following traumatic myofiber damage or temporal progression of myopathy, these satellite cells become activated and readily proliferate, differentiate and give rise to myoblasts, which fuse with damaged myofibers or form new myofibers.

Fibrosis is often associated with the impairment of stem cell populations in tissues, which are observed in many disease conditions. In skeletal muscle, fibrosis was considered to be caused by dysfunctional satellite cells. Using a murine model of muscular dystrophy (MDX), Alexakis and others reported the expression of collagen in primary myoblasts [46]. They have also found collagen expression in C2C12, a myoblast cell line. These findings indicate that satellite cells and transitionally amplifying myoblasts might deviate from their myogenic process to lead fibrosis-dominated degeneration. Furthermore, Keller [47] has suggested that the dysregulation of satellite cells during neonatal muscle growth is linked to rhabdomyosarcoma, a rare form of connective tissue tumor. Recently, in a mouse model of spinal muscular atrophy, mutation in the survival of motor neuron (SMN) gene is shown to affect the satellite cell’s intrinsic differentiation capacity, leading to a reduced efficiency in myotube formation [48]. Moreover, the conversion of satellite cells from a myogenic lineage to a fibrogenic lineage is documented in aging [49] and suggested that in aged mice, activation of the canonical Wnt signaling pathway is responsible for a pro-fibrotic phenotype. Other cases of fibrogenesis in myoblasts are also reported [50,51]. In addition, a recent study examining the stem cell function in aged people demonstrates that an age-related impairment of satellite cells is associated with increased co-localization of myostatin in satellite cells of type II myofibers [52]. Thus, tissue-specific stem cells responsible for regeneration, such as satellite cells in skeletal muscle, may also be involved in degenerative processes; however, whether the triggers for degeneration are cell autonomous or environmental influences, such as niche factors, is unknown.

#### Tissue-resident MSCs reside in the heart

In mammals, cardiac damage is not followed by the complete replacement of lost cellular components but is rather defined by a relatively minor capacity for regeneration and far more robust reparative response. Lacking an ability to regenerate, the formation of a scar in a timely manner following cardiac damage or during cardiac disease is critical in allowing continued organ functionality. Although there is a growing body of evidence demonstrating that the heart harbors its own population of TSCs, the cardiac stem cells [37,38], which account for the limited regenerative capacity of this organ, recent evidence has suggested that, similar to other organs, its repair processes may be governed by a cardiac-resident population of MSCs. The identification and elucidation of developmental origins of a novel population of stromal progenitors present within the myocardium has recently been reported [39], and further been demonstrated to be highly similar to MSCs derived from other tissues [40]. This population of cells contains all the fibroblastic colony-generating progenitors in the tissue, and was isolated based on markers essentially identical to those expressed by stromal progenitors in skeletal muscle (Sca-1^+^/PDGFRa^+^/CD31^–^) and was further shown to originate from the pro-epicardium. Expression of accepted markers of MSCs (CD44, CD90, CD29 and CD105) was confirmed on these cells, which also exhibited long-term growth potential in culture and were reported to possess the ability to form multiple mesodermal lineages (cardiomyocytes, endothelium, smooth muscle, adipocytes, cartilage and bone). As in skeletal muscle, in adult mice these cells occupy a perivascular, adventitial niche. A wider developmental potential encompassing elements of all germ layers has been reported for these cells upon co-transplantation with teratoma-forming ES cells, although the fact that fusion-induced reprogramming was not excluded in these experiments is a caveat. While the response of these cells to acute or chronic damage has yet to be analyzed in detail, it seems likely that similar to their phenotypically identical counterparts in skeletal muscle and other tissues [35,53], cardiac Sca1^+^, PDGFRα^+^ cells are a main source of fibrogenic cells in pathological cardiac fibrosis and that they participate in the formation of post-infarction scars.

#### Tissue-resident MSCs in the lung

In lungs, an anti-fibrotic role has been reported for exogenously delivered bone marrow derived MSCs, which likely rests on their ability to secrete trophic factors during normal regeneration. However, in keeping with what is reported in other tissues, lung derived/resident MSCs (LR-MSCs) have also been associated with fibrogenesis and aberrant tissue repair in lung injuries, such as transplantation surgery [54]. Lama and others first isolated LR-MSCs from the bronchoalveolar lavage fluid of lung transplantation patients [55]. These cells exhibited plastic adherence, formation of colony forming unit – fibroblasts (CFU-Fs), multipotency and expression of a combination of typical MSC surface markers CD44, CD73, CD90 and CD105 [55,56]. In most of the studies in which LR-MSCs exhibited a progressive fibrotic phenotype, investigators have used chronic injury models. Studies describing a positive role for exogenous MSCs, however, mainly relied on acute injury models, supporting the notion that these cells may play different roles in different settings.

It has been suggested that bi-directional crosstalk between stromal progenitors and cells involved in immune responses may control both the fate and function of LR-MSCs and vice versa. Jun and others recently characterized a population of lung-derived stromal cells (Hoechst^dim^/CD45^-^), which attenuated bleomycin-induced lung fibrosis and modulated local immune function by inhibiting antigen driven proliferative responses of effector T cells and decreasing the number of lymphocytes and granulocytes in bronchoalveolar fluid when transplanted [57]. These cells were distinct from lung fibroblasts in terms of gene expression, showing decreased expression of genes associated with inflammation, myofibroblast specification and extracellular matrix production. Unfortunately, as in other tissues, the heterogeneity of methods and markers used for the definition and characterization of LR-MSCs makes it very difficult to compare different studies and reach a consensus on their role at this time.

Apoptosis of stromal cells has been proposed to be one of the main mechanisms leading to the resolution of fibrosis during normal wound healing in many organs, and it is believed that in progressive fibrotic lesions, MSCs and their progeny escape its induction, leading to increased matrix deposition. Proposed roles of macrophages, T cells and the inflammatory microenvironment in general in regulating the survival of LR-MSCs in airway and interstitial pulmonary fibrosis are reviewed elsewhere [58].

### Alternative cellular sources for tissue-effector myofibroblasts

The progression from regeneration to repair invariably involves the development of fibrosis, defined as an excessive deposition of extracellular matrix. The principal cell type known to be involved in this process is an activated fibroblast derivative called a myo-fibroblast. The transition towards fibrosis was traditionally thought to involve expansion of stromal progenitors and subsequent differentiation into myo-fibroblasts, defined by increased synthesis of ECM proteins, such as fibrillar collagens and fibronectin as well as *de-novo* expression of alpha-smooth muscle actin. Although the importance of myofibroblasts in the development of fibrosis is generally accepted, there continues to be a significant debate whether alternative cellular sources, rather than differentiation from tissue-resident mesenchymal progenitors, exist for myofibroblasts.

The notion that collagen-producing myofibroblasts arise solely from the proliferation and differentiation of tissue-resident cells began to be questioned in the mid-1990s when two alternative cellular sources were proposed: (1) epithelial cells undergoing epithelial-to-mesenchymal transition (EMT) [59] and (2) circulating bone-marrow derived fibrocytes [5]. These concepts have important implications towards both the theoretical cellular processes underlying the development of fibrosis, and also the development of novel therapeutics to abrogate the process. Despite a wealth of literature supporting both theories, a growing number of recent studies employing much more rigorous lineage tracing analysis have cast a significant degree of doubt on the notion that fibrogenic cells arise from sources outside of tissue-resident MSCs.

#### Epithelial-to-mesenchymal transition (EMT)

Long known to be involved in metazoan embryogenesis, recent studies provided evidence that epithelial mesenchymal transition can also occur in adult tissues during the development of fibrosis as well as the progression and metastasis of cancer. Although the prevalence, as well as importance, of EMT in both embryogenesis and cancer is rarely disputed, a growing body of recent evidence has led many to reject the notion that EMT plays a role in solid organ regeneration and repair [60-62]. Recent use of more rigorous lineage tracing tools have strongly called into question the ability of epithelial cells to transition into collagen-producing mesenchymal cells during repair processes in numerous tissues, such as the kidneys [63-65], liver [66-68] and lungs [69]. There is a growing consensus that although EMT can be achieved *in vitro* through transforming growth factor (TGF)β1 treatment, this process does not make any significant contribution *in vivo* during tissue repair. Additionally, research attributing EMT as an important source of myofibroblasts have widely used the marker FSP1 (also known as S100A4) as a marker of epithelium-derived fibroblasts, which has repeatedly been shown to not label collagen producing cells in some tissues [65] and to lack specificity by labeling other cells, such as monocytes, macrophages, neutrophils and granulocytes [70]. Such critiques towards the field of EMT in tissue repair can also be applied to other processes, such as endothelial-to-mesenchymal transition (EndMT), which has used similarly questionable methods to demonstrate EndMT as an important source of tissue-effector myofibroblasts [71]. Further, additional doubt can be cast on EMT and EndMT data due to recent reports which demonstrate that Cre drivers previously thought to exclusively label epithelial or endothelial lineages (for example, Tie2) do not possess the necessary specificity to conclude that progeny labeled by these markers are exclusively derived from the epithelium or endothelium [41].

#### Fibrocytes

Circulating bone marrow derived mesenchymal progenitors, termed fibrocytes, have been proposed as a second alternative source of collagen-producing myo-fibroblasts in situations of tissue repair [5]. First described in 1994 [72], fibrocytes have classically been identified using a combination of hematopoietic markers CD34 and CD45, as well as the mesenchymal markers vimentin and collagen 1, although numerous further markers have been added in recent years [73]. Although there continues to be numerous studies published describing the role of circulating cells in the development of fibrosis, a number of recent reports have begun to call into question this model. Central to arguments that oppose the role of fibrocytes in fibrosis has been that much of the data supporting this model is based on phenotypical identification of fibroblasts of bone marrow derived origin, rather than characterization of the functional role these cells have in the development of tissue fibrosis. Following this, more recent studies employing more sophisticated techniques, such as genetic polymorphisms of collagen proteins in sex mismatched transplant recipients [74], as well as more specific collagen transgenics [75], have provided compelling evidence that, in numerous types of tissue repair, collagen-producing myofibroblasts arise solely from cells residing within the organ. Additionally, it should be noted that the same problematic reagents employed to identify the role of EMT in fibrosis, such as fibroblast markers of dubious specificity (for example, FSP1, vimentin) are also prevalent in a number of studies examining the role of fibrocytes [71].

### Signaling in MSCs

**Figure 3:**
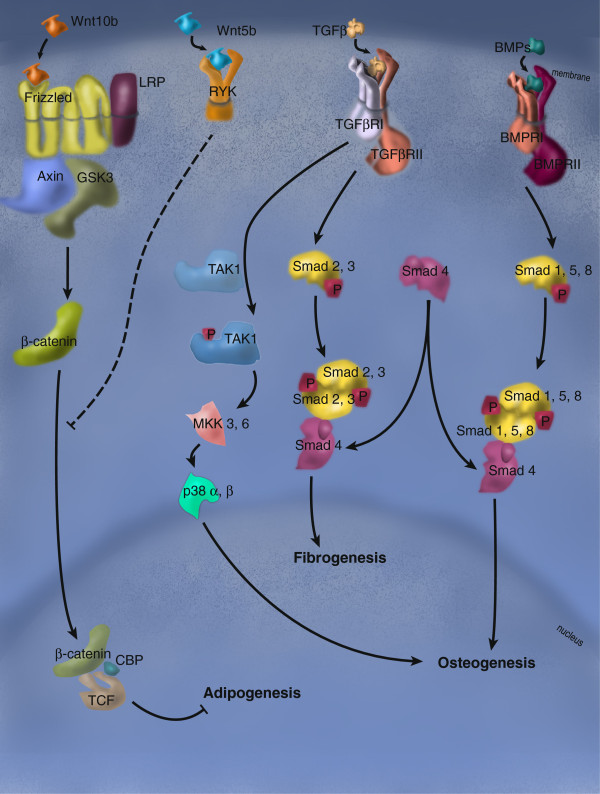
**Signaling pathways driving mesenchymal stem cells to differentiate into lineages found in reparative disorders.** While Wnt10b represses adipogenesis through the activity of B-catenin/TCF/Lef transcriptional complexes, activation of the non-canonical Wnt pathway by Wnt5b leads to repression of TCF/β-catenin transcriptional activity and yields the opposite results. Non-canonical TGFβ signaling participates in bone formation through activation of the osteogenesis regulator Runx2. On the other hand, canonical TGFβ signaling plays a central role in the regulation of the fibrogenic gene program. The BMP signaling pathway drives osteogenesis through SMADs 1, 5, 8 and shares the SMAD4 component with the TGFβ pathway.

Given their relevance in tissue regeneration, MSCs must maintain fluid communication with their surroundings. Indeed, a variety of stimuli, including physical and chemical signals originating in both the niche and the systemic environment, convey information to the MSCs. Integration of such signals can result in alteration of the otherwise quiescent state of MSCs, eliciting a sequence of fate choices that may include proliferation, self-renewal, migration, differentiation and cell death. In the absence of tissue damage and inflammation, systemic and metabolic cues can modulate the activity of stem cells under what can be regarded as homeostatic conditions [76]. Upon tissue damage, however, acute signals become the leading cues directing MSC activity. The combination of systemic and acute stimuli eventually drives the fate choice of MSC-derived progenitors into lineages that will contribute to the regeneration of the tissue. Under pathological conditions, however, aberrant signaling can lead to the development of ectopic cell types that contribute to the degeneration of the damaged tissue. In addition to the better-characterized pathways, such as FGF, PDGF and EGF, current advances in the study of the TGFβ, BMP and Wnt signaling cascades have disclosed a critical role for these factors in the regulation of mesenchymal stem cell behavior during tissue regeneration. The fact that the three pathways interact closely, partly through shared intracellular components, provides a number of interesting signaling crossroads that will be worth exploring in further depth. A summary of these signaling pathways and their effects is illustrated in Figure 3.

TGFβ, a member of the TGFβ superfamily, constitutes one of the major regulators of mesenchymal fate choice in postnatal life [77]. TGFβ signaling supports the early stages of chondroblastic and osteoblastic differentiation, while acting as an inhibitor of the advanced stages of osteoblast differentiation [78]. TGFβ inhibits adipogenic differentiation [79] through a route that involves interaction between the canonical complex SMAD3/SMAD4 with the transcriptional regulator C/EBP [80].

TGFβ signaling plays a pivotal role in both dermal homeostasis and hair follicle regeneration, where TGFB2 produced by the dermal papillae of the follicles drives hair follicle stem cells out of quiescence and activates them during the telogen-to-anagen transition [81]. Timely TGFβ release is critical for the initial stages of wound healing. Following damage, TGFβ1, -2 and −3 are secreted by various cell types, including platelets, fibroblasts, macrophages and keratinocytes. TGFβ signaling stimulates the temporary production of extracellular matrix (ECM) by fibroblasts and attracts macrophages that will participate in the inflammatory response [82]. Aberrant TGFβ signaling in the dermis, on the other hand, elicits excessive ECM deposition, fibrosis and scar formation that can lead to the formation of skin keloids [82]. In addition to its effect on fibroblasts and macrophages, TGFβ stimulates proliferation and sphere colony formation in skin-derived precursors (SKPs) *in vitro*, without altering their multipotency [83]. Excessive TGFβ production also correlates with skeletal muscle (SM) fibrosis [84], a characteristic feature of Duchenne muscular dystrophy [85]. Within SM, TGFβ targets mesenchymal Lin^-^/α7^-^/Sca-1^+^/PDGFRa^+^ progenitor cells that reside in the interstitial mesenchyme and can differentiate into collagen-producing fibroblasts [35]. Importantly, the same progenitor population can adopt the adipogenic lineage upon SM degenerative damage, leading to intramuscular ectopic fat accumulation [19,20,31].

The TGFβ family of signaling proteins is also important in the maintenance and expansion of bone and cartilage, largely through BMP proteins and TGFβ itself [76]. TGFβ promotes the proliferation, early differentiation and lineage commitment of bone progenitors through Smad2/3 and TAK1-MKK-p38 signaling [78]. Members of the BMP: BMP-2, 4, 5, 6 and 7 constitute osteogenic inducers. In particular, BMP-2 expression is sufficient for full osteogenic commitment, and loss of BMP-2 leads to impaired osteogenesis [86]. BMP-2 signals through type -I and -II BMP receptors and through the ALK2 receptor, leading to the activation of the Smad1/5/8 canonical pathway [87,88]. Following TGFβ and BMP induction, Smad and MAPK signals converge to regulate the activity of Runx2, a master transcriptional regulator that commands the expression of the osteogenic gene program, via Dlx5 [77]. In addition, Dlx5 also activates Osterix, a regulator of osteoblast maturation, independently of Runx2 activation [89].

Importantly, both the TGFβ and the BMP pathways connect with other signals that participate in bone formation. Components of the TGFβ signaling pathway interact with components of the pituitary hormone (PTH), Wnt and fibroblast growth factor (FGF) signaling pathways [90]. The BMP pathway, on the other hand, cross-talks with Notch, FGF and Wnt signaling [90]. Aberrant BMP signaling has been linked to heterotopic ossification, a pathological condition characterized by bone formation in skeletal muscle and soft tissues [40]. Mutations in the regulatory domain of the Alk2 receptor leading to hyper activation of the BMP signaling pathway have been shown to mimic the pathophysiology of fibrodysplasia ossificans progressive (FOP), an extreme form of heterotopic ossification [91]. Recent advances in the identification of the cellular substrate of FOP indicate that a muscle-resident Lin^-^/Sca-1^+^/PDGFRa^+^/Tie2^+^ mesenchymal cell population can also adopt the osteogenic lineage upon induction with BMP2 [41]. Consistent with the previously discussed role of Lin^-^/Sca-1^+^/PDGFRa^+^ cells in SM fibro-fatty degeneration, the Lin^-^/Sca-1^+^/PDGFRa^+^/Tie2^+^ cells also generated ectopic adipocytes in the lesions induced by BMP2 injection [41].

The Wnt family comprises secreted cysteine-rich glycopeptides that act in a paracrine and autocrine manner through a so-called canonical B-catenin-dependent and/or a non-canonical B-catenin-independent pathway. MSCs have been reported to express several Wnt ligands, including Wnt2, Wnt4, Wnt5a, Wnt11 and Wnt16, along with Wnt receptors of the Frizzled (FZD) family FZD2, 3, 4, 5 and 6, and co-receptors including LRP5 and 6. While the contribution of B-catenin-independent signaling on MSC activity is poorly understood, B-catenin-dependent signaling has been shown to play an important role in adipogenic and osteogenic differentiation of MSCs. Binding of Wnt to the FZD/LRP receptor complex induces the dissociation of the Axin/APC/GSK3B complex which, in the absence of Wnt signaling, phosphorylates B-catenin leading to its ubiquitination and degradation. Upon stabilization, B-catenin translocates into the nucleus, where it interacts with transcription factors of the lymphoid enhancer-binding factor/T-cell-specific factor (LEF/TCF) to induce the transcription of Wnt-regulated genes. Wnt molecules participate in adipogenic differentiation via the canonical B-catenin pathway. Wnt10b maintains preadipocytes undifferentiated by blocking the activity of the pro-adipogenic factors C/EBPα and PPARγ [92,93]. Indeed, transgenic mice overexpressing Wnt10b under the FABP4 promoter possess less adipose tissue in regular diet conditions and are resistant to diet-induced obesity. Those data were confirmed independently by overexpression of a dominant-negative form of TCF4 that facilitated adipogenic differentiation. Wnt5 also blocks PPARγ function, through a mechanism that involves H3K9 methylation [94]. In contrast to their inhibitory effect on adipogenesis, Wnt molecules induce osteogenic differentiation of MSCs. Osteogenic differentiation of MSCs - via a non-canonical pathway - with concomitant inhibition of adipogenic mechanisms has been shown to occur in MSCs [95,96]. The dual role of Wnt5 has also been shown *in vivo*, in the above-mentioned FABP4-WNT10B transgenic mice, in which the reduction in fat tissue is accompanied by an increase in bone mass, and reduced bone loss [97]. Altogether, a pivotal role for the Wnt family can be proposed by which these molecules regulate the balance between adipogenic vs. osteogenic lineage in MSCs.

## Conclusions

Reparative disorders are commonly accompanied with tissue injuries and subsequent repair processes. Stem cells are extremely important for tissue repair either by differentiating into new cells to replace damaged tissue (TSCs) or to aid in the regenerative or reparative process (MSCs). During the past decade, employment of various isolation and lineage-tracing methods both *in vivo* and *in vitro* has led to the identification of several types of adult tissue resident stem cells in distinct organs, and of a phenotypically homogeneous population of stromal progenitors present in all tissues analyzed and likely to be the *in vivo* equivalent of the ill-defined but often mentioned “mesenchymal stem cell”. However, the draw back in many studies has been the presence of functional heterogeneity within stem cell populations, which hinders the generalized characterization or comparison of these cells across species and tissues to use in therapeutic settings. Overcoming this obstacle will likely require high throughput single cell analysis techniques that are just starting to be available. Apart from their well-established role in regeneration, TSCs may also contribute to fibrosis, fatty degeneration or heterotopic ossification. To what extent this happens, however, is as yet unknown, and overwhelming evidence implicates MSCs as the main culprits for most reparative disorders. In addition, recent findings fundamentally challenge the hypothesis that MSCs also derive from EMT or EndMT, and the existence of circulating fibrocytes, suggesting that local progenitors are the main cell type involved in repair. Although MSCs’ participation in reparative disorders is proven, the molecular mechanisms by which they control the reparative process and regulate other cell types involved is critical for therapeutic intervention and in turn alter the fate choices of MSCs during repair. Promisingly, tissue resident MSCs have the potential to be included in cell-based therapies to treat reparative disorders as alternative autologous cell sources. Moreover, TGFβ, BMP and Wnt signaling cascades are considered as key communication partners with other known signaling molecules in the regulation of MSCs and, therefore, viewed as potential therapeutic targets. To fruitfully deploy modulators of these pathways, however, the complexity of interaction of MSCs both at cellular and molecular levels need to be further elucidated.

## Abbreviations

CFU-Fs: Colony forming unit – fibroblasts; ECM: Extracellular matrix; EMT: Epithelial-mesenchymal transition; EndMT: Endothelial-mesenchymal transition; FAPs: Fibro-adipogenic progenitors; FGF: Fibroblast growth factor; FZD: Frizzled; LEF/TCF: Lymphoid enhancer-binding factor/T-cell-specific factor; LR-MSCs: Lung derived/resident MSCs; MDSPCs: Muscle derived stem/progenitor cells; MSCs: Mesenchymal stromal cells; SKPs: Skin-derived precursors; SMN: Survival of motor neuron; TGF: Transforming growth factor; TSCs: Tissue-specific resident stem cells.

## Competing interests

The authors declare that they have no competing interests.

## Authors’ contributions

TP, DRL, BP and RHZ surveyed the literature and were involved in drafting the manuscript. RHZ was also responsible for formatting citations and the bibliography. FMR contributed through conception of content, supervision, correction and revision of the manuscript. All authors read and approved the final manuscript.
